# Buccally or Lingually Tilted Implants in the Lateral Atrophic Mandible: A Three-Year Follow-Up Study Focused on Neurosensory Impairment, Soft-Tissue-Related Impaction and Quality of Life Improvement

**DOI:** 10.3390/medicina59040697

**Published:** 2023-04-02

**Authors:** Iulian Filipov, Lucian Chirila, Federico Bolognesi, Corina Marilena Cristache

**Affiliations:** 1 Doctoral School, “Carol Davila” University of Medicine and Pharmacy, 37 Dionisie Lupu Street, 020021 Bucharest, Romania; iulian.filipov@drd.umfcd.ro; 2 Department of Maxillofacial Surgery, “Queen Maria” Military Emergency Hospital, 9 Pietii Str., 500007 Brasov, Romania; 3 Department of Oral and Maxillofacial Surgery, “Carol Davila” University of Medicine and Pharmacy, 19 Plevnei Ave., 010221 Bucharest, Romania; lucian.chirila@umfcd.ro; 4 Oral and Maxillo-Facial Surgery Unit, IRCCS Policlinico di Sant’Orsola, Via Giuseppe Massarenti, 9, 40138 Bologna, Italy; 5 Department of Biomedical and Neuromotor Sciences (DIBINEM), University of Bologna, 59 Via S. Vitale, 40100 Bologna, Italy; 6 Department of Dental Techniques, “Carol Davila” University of Medicine and Pharmacy, 8, Eroilor Sanitari Blvd., 050474 Bucharest, Romania

**Keywords:** alveolar ridge, atrophy, dental implants, quality of life, mandibular nerve

## Abstract

*Background and Objectives:* In the severely resorbed posterior mandible, implant placement requires either bone regenerative procedures, subperiosteal implants or short implant placement with drawbacks including morbidity and increased treatment costs and duration. To overcome these inconveniences, some unconventional alternatives have been suggested, such as buccally or lingually tilted implants in the lateral mandible, bypassing the inferior alveolar nerve. The aim of the present retrospective study was to evaluate the three-year survival rate of implants inserted in the posterior atrophic mandible, bypassing the inferior alveolar nerve. The assessment was focused on the occurrence of postoperative complications related to neurosensory impairment and soft tissue impaction, as well as overall improvement in quality of life. *Materials and Methods:* Patients with severe bone atrophy in the lateral area of the mandible were included in the present study. Only the implants tilted either buccally or lingually to bypass the inferior alveolar nerve were analysed. The relation between peri-implant soft tissue and the healing abutment was assessed and a secondary revision surgery was performed when indicated. The Semmes–Weinstein pressure neurological test was used for qualitative assessment of inferior alveolar nerve function and the Geriatric Oral Health Assessment Index (GOHAI) was used for evaluating Oral-Health-Related Quality of Life (OHRQoL). *Results:* Fourteen implants were placed in nine patients during the evaluation period. Survival rate was 100%, temporary paraesthesia occurred in one patient and a limited definitive paraesthesia was seen in another patient. Mild or significant discomfort related to soft tissue impaction with healing abutment was observed in six out of nine patients. A statistically significant OHRQoL improvement was observed in all patients. *Conclusions:* Despite the limited number of patients and observation time, insertion of implants buccally or lingually bypassing the inferior alveolar nerve is a predictive treatment option for patients with severe bone atrophy in the posterior mandible.

## 1. Introduction

The presence of sufficient bone volume is a prerequisite criterion for implant-prosthetic rehabilitation. 

In the severely resorbed posterior mandible, implant placement requires either bone regenerative procedures or short implant placement [1,2,3,4,5]. However, in many cases neither of the two options are feasible, due to local or systemic conditions specific to the patient in need [6]. Drawbacks of bone grafting include morbidity, and increases in treatment costs and duration. In order to overcome these inconveniences, some authors have suggested unconventional alternatives, such as placing the implants in a buccal or lingual fashion with respect to the inferior alveolar neurovascular bundle [7,8,9]. This approach has several advantages: minimally invasive procedure, less time consuming, short treatment time, and suitable for patients with relative or absolute contraindication for bone augmentation procedures.

However, these unconventional and technically sensitive procedures are not without associated risks, and alveolar nerve injury is a major concern during implant placement. Paraesthesia of the mental nerve was also described by several authors [8,9]. Soft tissue proliferation toward the prosthetic components is another complication especially in cases with high insertion of the buccal mucosa or when the implants are placed in a more posterior position [10]. 

When referring to tilted implant insertion in the posterior mandible, most published studies focused on implants survival [11] or success [12]. To our knowledge there is no study objectively investigating the occurrence and evolution of neurosensorial complications as well as soft-tissue-related impaction type complications, and no therapeutic options have yet been suggested for the latter.

The aim of the present retrospective study was to evaluate the three-year survival rate of implants inserted in the posterior atrophic mandible, bypassing the inferior alveolar nerve. The assessment was focused on the occurrence and evolution of postoperative complications related to inferior alveolar nerve neurosensory impairment, the soft tissue impaction related complication rate, as well as the overall improvement in quality of life.

The null hypothesis of the present study was formulated as follows: buccally or lingually tilting will not influence the survival rate of the dental implants.

## 2. Materials and Methods

This study was pursued in compliance with the World Medical Association Declaration of Helsinki, the Belmont report, the Council for International Organizations of Medical Sciences (CIOMS) guidelines, and the International Conference on Harmonization in Good Clinical Practice (ICH-GCP). The investigation was conducted as a retrospective study on a sample of 9 patients treated from 2016 to 2019 in Brasov, Romania and approved by the Institutional Ethics Committee of “Queen Maria” Military Hospital, Brasov, Romania, no. 1313/2022. Moreover, written consent from each subject was obtained. The selected patients had either local or systemic contraindications to bone grafting procedures or short implant placement. All participants met the following inclusion criteria: (1) age > 18 years old, (2) no contraindication for dental implant placement, (3) replacement of one or several teeth in the posterior region of the mandible, (4) class 5 or 6 atrophy in the lateral mandibular zone according to Cawood & Howell classification [13], (5) a minimum of 5 mm of bone lateral to the mandibular channel, (6) all the patients were treated by the same surgeon (I.F.) using the same surgical protocol, and (7) at least one of the placed implant should be tilted buccally or lingually bypassing the inferior alveolar nerve. All patients gave their written informed consent for the treatment protocol. The prosthetic rehabilitation was either an overdenture or a fixed cemented restoration. A Cone Beam Computed Tomography (CBCT) was performed before treatment to assess the residual bone volume and to evaluate the position of the inferior alveolar nerve. The implants placed were MegaGen AnyRidge^®^ (MegaGen, Daegu, Republic of Korea).

### 2.1. Surgical Technique

The surgical procedures were performed under local infiltrative anaesthesia into buccal and lingual alveolar mucosa, according to a previously described technique [6]. No anaesthesia into the Spix spine was done in order to maintain sensorial feedback from the patient. A preoperative rinsing of the oral cavity with a 0.2% Chlorhexidine (Dentosan^®^, Recordati SpA, Milano, Italy) antiseptic solution performed. Antibiotic prophylaxis was begun 1 h before surgery, with 2 g of amoxicillin clavulanate (Augmentin^®^, GlaxoSmithKline, London, UK) [14,15]. 

**Figure 1:**
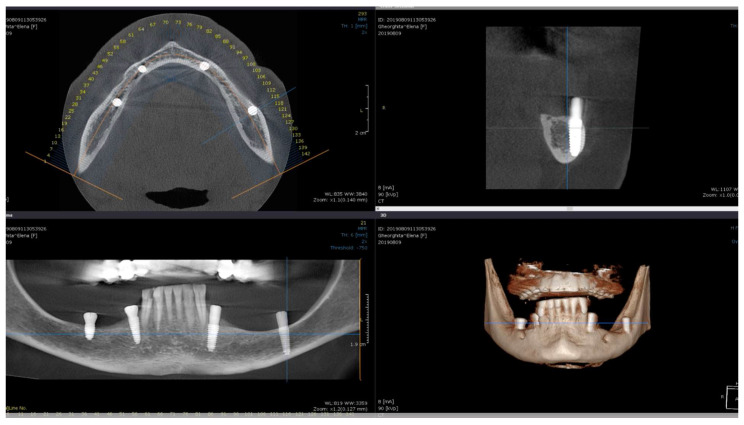
Postoperative CBCT of patient no. 6: (**Upper left**)—axial view of the mandible; (**upper right**)—sagittal left view of the buccally tilted implant (37); (**lower left**)—coronal view of the mandible; (**lower right**)—3D view of the mandible.

**Figure 2:**
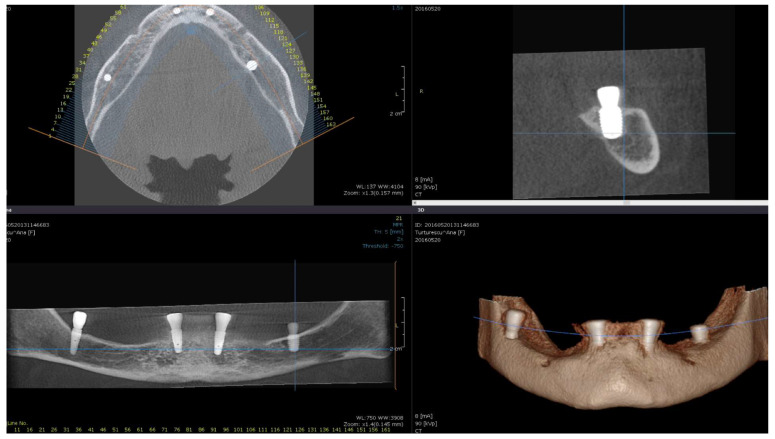
Postoperative CBCT of patient no. 5: (**Upper left**)—axial view of the mandible; (**upper right**)—sagittal left view of the lingually tilted implant (36); (**lower left**)—coronal view of the mandible; (**lower right**)—3D view of the mandible.

Direct visual access to the residual bone was obtained by elevating a full muco-periosteal flap. A lance drill was used to mark the implant site at approximately 2 mm towards the midcrest with respect to the buccal or lingual cortex. To avoid thermal osteonecrosis, copious cooling irrigation, new drills, and a maximum speed of 800 RPM were used for implant site preparation. After the osteotomy site was marked, a pilot 2 mm drill was selected to perform the preparation for the length of the selected implant. In-and-out manoeuvres were performed in order to facilitate cooling of the drills. If the implant is planned to be placed buccally, the drill must be oriented with the apex faced towards buccal wall, trying to keep the axis of the drill parallel to the buccal cortex. In most of the cases, if the pilot drill direction was properly assessed, minimal or even no bleeding will be observed and the patient will indicate no pain. If the osteotomy preparation was performed buccally, a bone dehiscence might be present. In this scenario, the following drills must be slightly pressed towards the inner wall (wall facing towards the middle of the alveolar crest) of the osteotomy site. This manoeuvre is performed by a digital propelling force on the head of the handpiece, according to the preparation protocol for D1 bone recommended by the implant company. If a 4.0 mm diameter tapered implant (AnyRidge, Megagen Implant Co., Daegu, Republic of Korea) is planned to be used, the following drills were selected for convenient site preparation: 2.7 mm, 3.3 mm, 3.8 mm for the whole length of the osteotomy and a 4.3 mm drill was used only for countersink. Implant insertion was done with the surgical motor (at 35 RPM). A similar drilling protocol is applied for 3.5 mm diameter implants (last drill 3.3 mm and 3.8 mm countersink) and a 4.3 mm drill for the whole length, for 4.5 mm diameter implants. Depending on the thickness of the surrounding soft tissues and the relation with the buccal mucosa, either a 7 mm long healing abutment (I-Gen Kit, Megagen, Daegu, Republic of Korea) or a customized healing abutment was connected to the implant, with digital clockwise rotations. Double layer suture (horizontal mattress and simple suture) was done with 5/0 resorbable suture (Vicryl, Ethicon, New Brunswick, NJ, USA). Patients were prescribed antibiotics (Augmentin^®^—amoxicillin plus clavulanic acid 1 g/every 12 h) and one tablet of anti-inflammatory drug (Ibuprofen 600 mg at every 8 h) for the following 5 days. Patients were instructed to avoid brushing the neighbouring teeth in the treated area and chlorhexidine solution (0.12%) was prescribed for daily usage (twice a day for 1 min). Sutures were removed after 14 days [16]. All patients were recalled for clinical checkup 1 day, 1 week, 2 weeks, and 2, 6, 12, 24, and 36 months after surgery. All complications, both during the surgery and postoperative, were reviewed. A postoperative CBCT was done to assess implant positioning. Buccally tilted and lingually tilted implant positioning are presented in Figure 1 and Figure 2, respectively.

### 2.2. Assessment of Neurosensory Disturbance (NSD)

An objective assessment of inferior alveolar nerve hypoesthesia/anesthesia was done by applying the Semmes–Weinstein (SW) pressure neurological test [17,18], adapted for the maxillofacial region [9,19]. The Touch-Test™ Sensory Evaluator (North Coast Medical, Inc., Morgan Hill, CA, USA) full kit, including 20 single-fiber (monofilament) nylon threads, was used for the evaluation. The tester-calibrated fibers are identified by values ranging from 1.65 to 6.65, generating reproducible buckling stresses ranging from 0.008 to 300 g. Higher monofilament values indicate greater monofilament stiffness (higher pressure for lower sensitivity). The test was used for qualitative assessment of inferior alveolar nerve function prior to surgery and 1 day after surgery. If any impairment of the alveolar nerve was detected, the test was repeated at 2 weeks, 2 months, 6 months and 12 months postoperatively in case of long persistence of the neurosensory disturbances. 

Testing was done as described in similar studies [19,20,21], with the patient sitting with their eyes closed, and the test areas were selected in random order. For an objective standardization, the anatomic area corresponding to the inferior alveolar nerve was divided into 3 areas:

1. Lower lip vermilion
2. Medial section of the chin: starting from a cutaneous midline corresponding to the midline of the chin and extending laterally to a vertical line that divides the lower vermilion into 2 equal segments.
3. Lateral section of the chin: starting from the vertical line that divides the lower vermilion into 2 equal segments and extending laterally to a vertical line corresponding to the commissure.

The lower border for the 2nd and 3rd designated areas was represented by a cutaneous line corresponding to the inferior margin of the mandible.

The tester monofilaments were applied four times in each of the three areas of interest, in ascending order of stiffness. 

The stimulus response was considered positive when there were at least 3 (75%) correct answers (3 of 4 correct stimuli). Patients were considered to have NSD if they did not respond postoperatively or they responded to only those filaments that were stiffer than those to which they responded preoperatively. 

Full recovery was considered when test was positive with the filament with equal stiffness than preoperatively.

Permanent neurosensory disturbance was defined as abnormal or negative clinical test results obtained at least 12 months after surgery. 

### 2.3. Healing Monitoring before the Prosthetic Phase

Several variables were assessed during pre-prosthetic healing period: swelling at the surgical site or adjacent lymph nodes, redness, bleeding, exudate, pain, malodour. A pink or red persistent (longer than 6 weeks) exudate was related to the presence of red blood cells (bleeding or blood-stained exudate) due to capillary damage which is usually trauma-induced [22,23]. In symptomatic patients, the trauma was related to soft tissue impaction by the healing abutment. Another objective sign of soft tissue impaction was light bleeding when the adjacent peri-implant soft tissues were touched with a periodontal probe. Other linked objective signs of inflammation were swelling and redness of the adjacent soft tissue. Rarely, submandibular lymph nodes were appreciated as swollen and painful on palpation. 

### 2.4. Soft Tissue Revision Surgery

**Figure 3:**
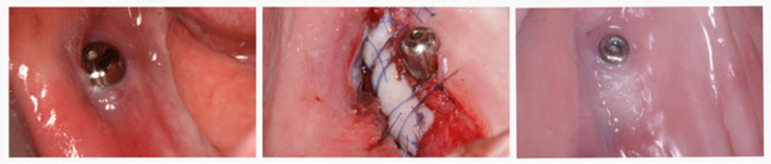
Patient no. 5 requiring revision surgery due to buccal impaction of the healing abutment (**left**); free gingival graft surgery (**middle**); postoperative healing (**right**).

The revision surgery for reconstruction of keratinized mucosa around the implant with subsequent muscular reposition and free gingival graft was proposed as a therapeutic remedy for patients with discomfort or peri-implant soft-tissue-related pain due to impaction with the healing abutment. The decision to perform the procedure was established not sooner than 2 months in order to assess the biological capacity of the peri-implant soft tissue to adapt to the newly created conditions (Figure 3).

### 2.5. Prosthetic Phase

Once the implants were assessed as well integrated, the patients were asymptomatic, and the soft tissues status was evaluated as optimal, the implant-supported restorations were placed. Either an overdenture or final fixed prosthesis was delivered [6,24].

### 2.6. Oral-Health-Related Quality of Life (OHRQoL) Assessment

The Geriatric Oral Health Assessment Index (GOHAI) consisting of 12 questions, developed by Atchinson and Dolan [25], validated for the Romania language by Murariu et al. [26] was administrated twice for each patient. Firstly, it was applied as a self-assessment interview during treatment planning, before implant surgery (baseline); and secondly, the questionnaire was administrated four months after prosthetic restoration (post-treatment). For both questionaries, the reference period used was past three months, according to the original version proposed by Atchinson and Dolan [25]. Each of the 12 GOHAI items has a set of possible answers distributed on a Likert scale (all the time = 5, very often = 4, fairly often = 3, sometimes = 2, seldom = 1, and never = 0). In the Romanian version of GOHAI, questions 3, 5, and 7 were reformulated as negatively worded questions, different from the original version, for an easier approach to the questionnaire such that it included only negatively worded questions. In this study, the total score was analyzed, with a value ranging from 0 to 60 with a lower score meaning a better quality of life. The GOHAI-Ro items are [27]:

1. Limit the kinds of food
2. Trouble biting or chewing
3. Problems swallowing comfortably
4. Problems speaking clearly
5. Discomfort when eating any kind of food
6. Limit contact with people
7. Unsatisfied with look of teeth
8. Used medication to relieve pain
9. Worried about teeth, gums or dentures
10. Self-conscious of teeth, gums or dentures
11. Uncomfortable eating in front of others
12. Sensitive to hot, cold or sweet foods

### 2.7. Data Analysis

All data were synthetized in Excel tables and compared and analyzed using OriginLab Pro 2021 software (Northampton, MA, USA). Kaplan–Meier survival analysis was used to determine whether the distribution of time-to-event (NSD or soft tissue impaction) or failure differed based on implant tilting (buccal or lingual), implant diameter, implant length, or implant position (premolar, first, second or third molar). Censoring was considered when no event or failure occurred during the observation period or the patient dropped out of the study [28]. 

Log-rank tests were conducted to determine whether the survival/complication distribution differed according to implant tilting (buccal vs. lingual), implant diameter and length, implant position, or type of restoration. 

Mann–Whitney U tests were used to investigate changes in OHRQoL after treatment.

Statistical significance was set at a *p* value < 0.05.

In order to describe the NSD status and the peri-implant soft tissue condition, descriptive analysis was used.

## 3. Results

The study population consisted of nine subjects, mean age 65.7 years old (±5.01) of which eight were women. Fourteen dental implants were inserted in the lateral area of the mandible on the position of second premolar (7.14%), first and second molar (50% and 35.72%, respectively), or even in a more posterior position than third molar (7.14%). Eleven implants were buccally tilted (78.57%) and three implants were lingually tilted (21.43%). 

No patients dropped out of the study during the evaluation period and no implant was lost at three years follow-up, leading to a 100% implant survival rate.

Table 1 shows the distribution of patients regarding age, gender, implant position, diameter and length, tilting direction, and type of prosthetic restoration.

**Figure 4:**
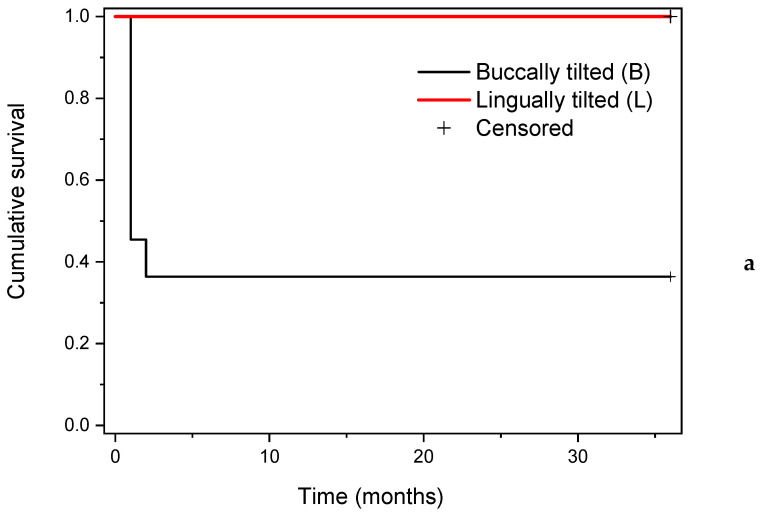

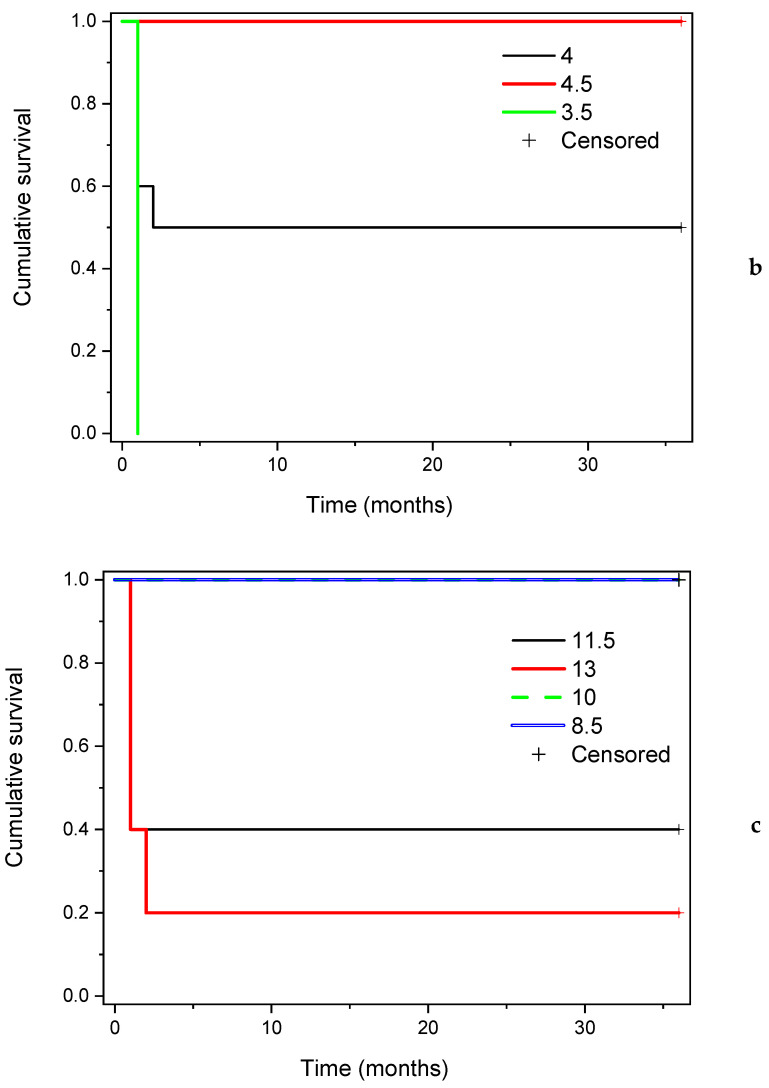

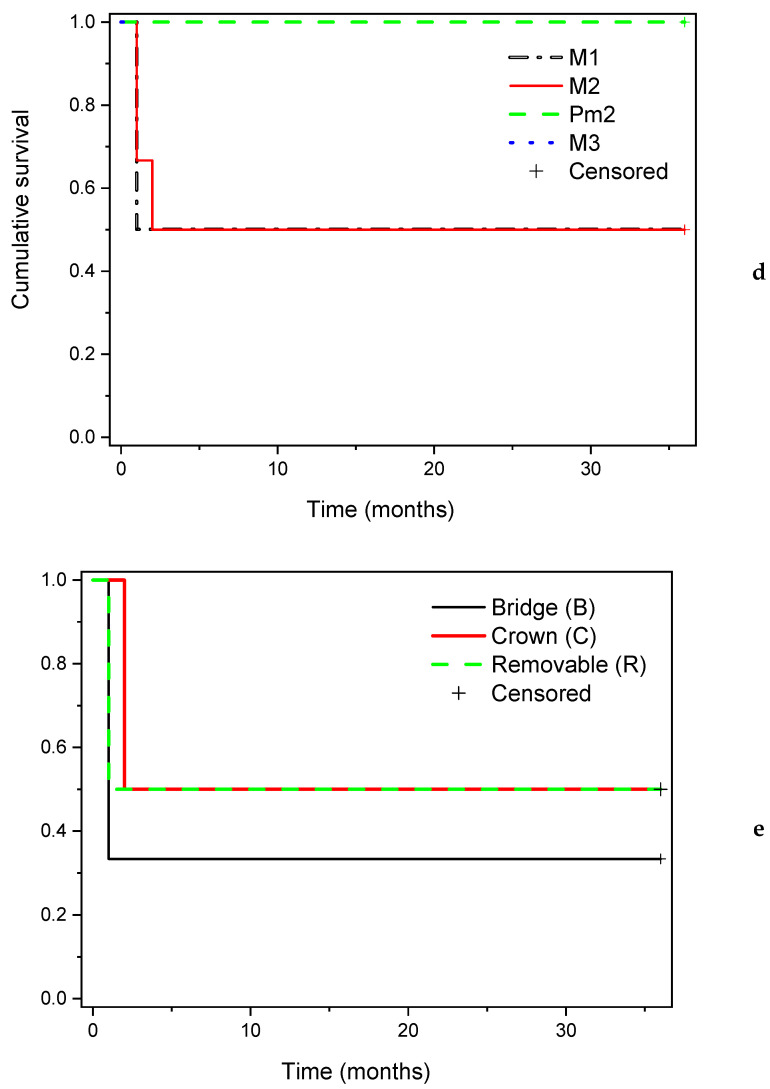
Implant cumulative survival rate according to (**a**) implant tilting (buccally or lingually); (**b**) implant diameter (in mm); (**c**) implant length (in mm); (**d**) implant position (M1—first molar, M2—second molar, Pm2—second premolar, M3—third molar); (**e**) type of prosthetic restoration (fixed—bridge or single crown, and removable—implant retained overdenture).

The three-year survival analysis regarding implant tilting (buccal vs. lingual), implant diameter, implant length, implant position (premolar, first, second or third molar), and type of prosthetic restoration is shown in Figure 4.

No statistically significant differences regarding implant survival related to implant tilting (log rank: χ^2^ = 3.22, df = 1, *p* = 0.07), implant diameter (log rank: χ^2^ = 4.23, df = 2, *p* = 0.12), implant length (log rank: χ^2^ = 5.20, df = 3, *p* = 0.16), implant position (log rank: χ^2^ = 2.07, df = 3, *p* = 0.56) or prosthetic restoration (log rank: χ^2^ = 0.34, df = 2, *p* = 0.84) were observed.

### 3.1. Assessment of Neurosensory Disturbance (NSD)

Seven patients (85.71%) with twelve implants sites showed a positive response when subjected to the tactile point pressure sensitivity test using the Semmes–Weinstein approach. Tactile point pressure neurosensitivity tests results revealed that 1 patient (patient no. 6 from Table 1) showed disturbances in all 3 regions, while a second patient (patient no. 1) showed NSD in the evaluated regions 1 and 2 at 24 h follow-up assessment. 

For these two patients with NSD, a specific treatment was prescribed:

- Orally administrated Prednisolone 5 mg (Sintofarm S. A., Bucharest, Romania) 5-day step-down dose (50-40-30-20-10 mg).
- B vitamins (Milgamma 100 mg, Worwag Pharma GmbH & Co, Stuttgart, Germany) for three weeks, three times per day.

The test revealed a restitution ad integrum of the neurosensory function after 2 months for patient no. 1, who initially showed NSD in 2 areas. For patient no. 6, who expressed neural impairment in all 3 regions, a slight residual NSD was evident at 12 months evaluation, persistent at 3 years follow-up.

### 3.2. Soft Tissue Impaction and Revision Surgery

A great number of patients (6 out of 9 or 66.7%) expressed mild or significant discomfort related to soft tissue impaction with the healing abutment. The location of the impaction was between the buccal peri-implant mucosa and the healing abutment and was observed at six molar sites (two—first molar, three—second molar, and one—third molar). No cases of lingually tilted implants were identified. In five patients (55.5% of all) soft tissue corrective procedures were performed. In four (80% of the surgically revised cases) patients, symptoms ceased to exist; while one patient reported a significant improvement, with some residual soft tissue disturbances during mastication. However, the patient developed a favourable mastication pattern within the following 7 months and expressed convenient satisfaction with the final outcome. 

### 3.3. Oral-Health-Related Quality of Life (OHRQoL) Assessment

Mean (SD—standard deviation) GOHAI total score at baseline (before treatment) was 44.89 (±6.37), and 17.67 (±3.74) four months after prosthetic restoration (post-treatment). Statistically significant overall improvements in all criteria assessed were recorded after final prosthetic restorations. All the patients, including patient no. 6, expressed high satisfaction with significant OHRQoL improvements.

## 4. Discussion

In the posterior mandible rehabilitation of patients with implants, supporting restoration in areas with severe bone resorption is a challenge in implant dentistry, mainly for elderly patients.

Bone regenerative techniques, such as distraction osteogenesis, block bone grafts or alveolar nerve lateralization are recommended for facilitating implant insertion in the posterior mandible. However, these techniques may involve risky surgery, increased healing time, increased cost and additional complications [29,30,31]. Moreover, for elderly patients, with compromised health, complex surgical procedures are often not indicated [6,32].

Short (less than 10 mm length) or extra short (4 mm length) implants could be a treatment option but require at least 7 mm residual bone above the inferior alveolar nerve [33].

Titanium subperiosteal implants with or without simultaneous bone morphogenetic proteins (BMPs) grafts have been proposed for atrophic mandibular bone preservation and overdenture retention [34]. However, their use involves invasive surgical procedures, frequent technical issues due to poor adaptation to the surgical site, and are prone to long-term complications [35]. The use of Computer Aided Design and Manufacturing (CAD-CAM) technology with 3D printing protocols could improve subperiosteal implants accuracy and reduce surgical procedures related to implant insertion [32], but cannot provide long-term bone preservation.

In the present study, we proposed the insertion of implants buccally or lingually bypassing the inferior alveolar nerve. This technique was described in detail in a case report and proof of concept on a patient with severe mandibular ridge atrophy and multiple comorbidities, limiting extensive surgeries [6]. The patent was restored with a removable bar retained overdenture and no complications occurred during the 14 months of follow-up. For the above-mentioned patient, four implants were inserted, two (3.5 mm × 7 mm implants, AnyRidge^®^, MegaGen, Daegu, Republic of Korea) anterior and two (3.2 mm × 10 mm implants, Mini^®^, MegaGen, Daegu, Republic of Korea) in the retromolar area, buccally bypassing the alveolar nerve [6]. For the above-mentioned case, the inferior alveolar nerve integrity was not affected, no additional soft tissue revision surgery was required and the OHRQoL assessed with the validated Oral Health Impact Profile for Edentulous Patients (OHIP-EDENT) questionnaire showed significant improvements in the patient’s quality of life.

In the present retrospective study, our aim was to objectively investigate the occurrence of complications related to inferior alveolar nerve neurosensory impairment or the soft tissue impaction, and whether these types of complications are related to buccally or lingually tilting of the implants.

Moreover, to our knowledge, there is no study investigating soft-tissue-related impaction type complications and no therapeutic options have yet been suggested. 

From the fourteen dental implants inserted bypassing the alveolar nerve, eleven implants were buccally tilted, and three were lingually tilted, due to the architecture of the residual alveolar bone at the specific site. The overall survival was 100%, with all the inserted implants functioning at three years follow-up. No statistically significant differences were found regarding implant survival when comparing tilting direction, implant diameter, implant length, implant position or prosthetic restoration. The null hypothesis of the present study could not be rejected.

Tilted insertion of dental implants, even > 45°, is a predictable and well-accepted procedure to eliminate distal cantilever [12], with long-term follow-up [8].

However, bypassing the alveolar nerve when inserting dental implants in the posterior mandible is a technically sensitive surgical procedure; but when well-planned, it has a predictable procedure, with a reduced number of complications. Only two cases of NSD occurred, one completely remitted after two months and one slightly persistent after three years, but without affecting the patients’ quality of life. These type of complications are not uncommon with other types of surgery such as transposition of the inferior alveolar nerve [31] or distraction osteogenesis [36].

Another complication was soft tissue impaction, occurring at six implants sites, all with buccally tilted implants; but only five sites required minor soft tissue revision surgery with free gingival graft (two months post implant insertion) and the obtained results were stable at three years follow-up.

All the patient expressed high satisfaction with the final outcome, with a significant improvement in quality of life.

The present retrospective study has some limitations, mainly regarding the low number of patients, mostly females, lack of a control group, and the limited follow-up period. Additionally, the surgical technique is challenging but could be more predictable and accessible with the use of digital planning, virtual reality (VR) and augmented reality (AR). Further randomized clinical studies are necessary to validate the treatment protocol. Buccally or lingually tilting dental implant insertions for atrophic mandibular rehabilitation could be a viable option for restoring patients’ functions and improving oral-health-related quality of life.

## 5. Conclusions

This retrospective observational study demonstrated that, even though a technically sensitive procedure was performed with a high risk for alveolar nerve impairment, most patients had their tactile neurosensory sensitivity totally preserved for 12 months after undergoing implant placement. A slight limited residual paraesthesia was assessed in one patient, without a subjective regress in quality of life. 

Soft tissue impaction type complication is a frequent complication that may be totally or partially resolved with vestibuloplasty and free gingival graft. 

Implant placement in limited bone volume—such as the cases previously outlined— necessitates certain skills, and significant variations related to the outlined results may exists.

However, for a safe and predictable dental implant insertion, an experienced oral surgeon, a CBCT investigation and precise planning, the existing residual bone volume, the degree of mouth opening, and the amount of mandibular atrophy are important factors to be considered.

Neurosensory disturbance may occur but is not uncommon for other recommended surgeries such as distraction osteogenesis or alveolar nerve transposition, and could be remitted with adequate postoperative treatment.

Despite the limited number of patients and observation time, the insertion of implants buccally or lingually bypassing the inferior alveolar nerve is a predictive treatment option for patients with severe bone atrophy in the posterior mandible. 

Further studies and the use of CAD-CAM-guided surgery may sustain the feasibility of this surgical approach. 

## Figures and Tables

**Table 1:** Distribution of patients.

Patient No.	Gender	Age	Implant Position	Implant Diameter/Length	Tilting Direction (B/L)	Loading Timing (Months)	Type of Restoration
1	F	71	36	4/11.5	B	6	bridge
			47	4/11.5	B	6	bridge
2	F	69	36	4/13	B	4	bridge
3	M	63	46	4/11.5	B	8	bridge
			36	4/10	L	8	bridge
4	F	58	47	4/13	B	4	crown
5	F	64	36	4.5/8.5	L	7	removable
			47	3.5/11.5	B	7	removable
6	F	61	37	4/13	B	7	bridge
7	F	74	35	4/10	B	3	bridge
			38	3.5/11.5	B	3	bridge
8	F	67	46	4/13	B	7	bridge
			47	4/13	B	7	bridge
9	F	65	36	4.5/8.5	L	4	crown

## Data Availability

Supplementary data are available on request from the corresponding author.
